# High burden of vitamin D deficiency in Chinese university students: impact of sun protection culture

**DOI:** 10.3389/fnut.2026.1889729

**Published:** 2026-07-28

**Authors:** Shikang Su, Simin Sun, Wenhao Ji, Huanhao Cai, Ziyang Lin, Wanlei Yang, Bin Fang, Yu Qian

**Affiliations:** 1 Department of Orthopedics Surgery, The First Affiliated Hospital of Zhejiang Chinese Medical University (Zhejiang Provincial Hospital of Chinese Medicine), Hangzhou, Zhejiang, China; 2 The First Clinical Medical College, Zhejiang Chinese Medical University, Hangzhou, Zhejiang, China

**Keywords:** bone metabolic markers, Chinese university students, skin tone preference, sun protection behaviors, vitamin D

## Abstract

**Background:**

Vitamin D serves a critical function in preserving human physiological well-being and is mainly synthesized within the skin through sunlight exposure. In China, the traditional cultural preference for fair skin is associated with widespread sun avoidance among many individuals, including university students, who seek to prevent skin tanning. Although sun protection habits and fair-skin pursuit are widely prevalent in China, their associations with vitamin D inadequacy have not been well clarified in the college student demographic.

**Methods:**

The present cross-sectional investigation was conducted from May to June 2025 in Hangzhou, using convenience sampling to enroll 174 regular university students and 17 outdoor-trained students as controls. Blood biochemical detection analyses were conducted to measure circulating 25(OH)D concentrations and bone turnover indicators, while standardized questionnaires were adopted to gather data on participants’ sun protection habits and skin color aesthetic preferences. Multiple linear regression models were constructed with adjustment for age, sex, BMI, daily sun exposure duration, physical activity level, and smoking and drinking status.

**Results:**

A total of 93.10% of the usual students had vitamin D deficiency (<50 nmol/L), among whom 59.20% were deficient (<30 nmol/L). These rates were considerably higher than those observed in outdoor students (29.41 and 23.53%, respectively). In the 174 regular university students, Pearson correlation analysis showed a significant negative association between sun protection behavior scores and serum 25(OH)D concentrations (*r* = −0.387, *p* < 0.001). This association remained significant after adjusting for age, BMI, sex, and sun exposure time in multiple linear regression models. Additionally, the study population generally exhibited a cultural preference for fair skin, which was also significantly associated with lower serum 25(OH)D levels.

**Conclusion:**

Chinese university students have a strikingly elevated prevalence of vitamin D insufficiency and deficiency, which is closely linked to excessive sun protection related to traditional fair-skin aesthetic norms. The present results reveal a notable contradiction between skin aesthetic pursuits and skeletal health status, suggesting that culturally tailored intervention strategies are essential for improving vitamin D levels among young adult populations.

## 1 Introduction

As a crucial fat-soluble vitamin, vitamin D exerts diverse physiological effects. It is fundamentally responsible for modulating systemic calcium and phosphorus balance, sustaining normal skeletal conditions, and protecting individuals against osteoporosis (1). Emerging epidemiological and clinical research consistently demonstrates that insufficient vitamin D status correlates strongly with increased vulnerability to various skeletal abnormalities, including hypertension, cardiovascular diseases, type 2 diabetes mellitus, and multiple cancer subtypes (2, 3). For most individuals worldwide, over 80% of endogenous vitamin D production occurs in the epidermis upon exposure to solar ultraviolet B (UVB) rays, with dietary intake and supplementation contributing only a minor fraction of the body’s total vitamin D requirement (4). Mounting evidence has acknowledged that inadequate solar exposure serves as a major modifiable contributor to the widespread occurrence of vitamin D insufficiency worldwide (5). Serum 25-hydroxyvitamin D [25(OH)D] serves as the gold standard biomarker for evaluating vitamin D nutritional status. Although no unified global standard has been established for optimal circulating 25(OH)D levels, our investigation adopted cutoffs consistent with the Institute of Medicine (IOM) consensus and widely used epidemiological criteria: vitamin D deficiency was categorized as serum 25(OH)D concentrations less than 30 nmol/L (12 ng/mL), while vitamin D insufficiency was defined using a threshold of less than 50 nmol/L (20 ng/mL) (6). Existing research indicated that vitamin D deficiency rates among the Chinese population are significantly elevated compared with those documented in other countries globally. Relevant population-based investigations indicated that among American adults, the percentage of subjects with serum 25(OH)D concentrations below 30 nmol/L reached around 5%, and the rate for levels under 50 nmol/L was 23.3%. By contrast, an extensive systematic review combined with meta-analysis reported corresponding figures of 20.7 and 63.2% among adult residents in China (7, 8).

While latitude, skin pigmentation and dietary intake influence vitamin D nutritional status, the striking disparity observed in vitamin D deficiency rates between China and Western countries suggests that the implications of social culture for health-related behaviors are important to consider. There exist obvious cultural disparities between China and Western countries; Chinese people—including college students—are more likely to have more sun protection behaviors to avoid skin darkening, given that pale skin has long been a deeply rooted cultural ideal in Chinese society. A research investigation conducted in Beijing illustrated the daily sun protection habits of local residents. The results showed 58.8% of participants regularly applied sunscreen as their main protective approach, followed by wearing sun-protective clothing at 49.3%, holding parasols at 45.4%, wearing sunglasses at 45.3%, and wearing sun hats at 42.2%. Meanwhile, over half of the interviewees, accounting for 52%, viewed skin tanning as unhealthy and aesthetically unappealing (9). Another study of sun protection behaviors among American adults found that shade seeking was the most common practice (37.1%), followed by sunscreen use (31.5%) and wearing ankle-length clothing (28.4%) (10). Although sun protection behavior represents an established risk factor for reduced vitamin D levels (11), the possible relationship between China’s unique sun protection cultural norms and vitamin D insufficiency, as well as its potential association with bone metabolism, has not been systematically studied in Chinese young college students. We hypothesized that the cultural preference for fair skin in China is associated with frequent sun protection behaviors among university students, which may be an independent correlate for vitamin D insufficiency, and such nutritional deficits may correlate with alterations in bone turnover markers (BTMs).

The present cross-sectional investigation set out two core research aims. The first aim was to characterize the distribution of circulating 25(OH)D concentrations and determine the prevalence rates of vitamin D deficiency and insufficiency among Chinese college students. The second objective sought to explore key determinants underlying the inadequate vitamin D status documented in this young population, with special emphasis on the influence of traditional Chinese aesthetic values favoring fair skin and the resultant widespread adoption of daily sun avoidance practices.

## 2 Materials and methods

### 2.1 Study design and participants

#### 2.1.1 Study design and rationale

This was an observational cross-sectional study conducted at Zhejiang Provincial Hospital of Traditional Chinese Medicine. A cross-sectional design was selected for two core reasons aligned with the study’s objectives: first, it enables accurate estimation of the prevalence of vitamin D insufficiency and deficiency in the target young adult population; second, it efficiently captures concurrent associations between sociocultural behavioral factors (sun protection habits, skin tone aesthetic preferences) and circulating biochemical markers, providing foundational empirical evidence for subsequent prospective interventional research.

All study procedures complied with the ethical guidelines of the Declaration of Helsinki, and the research protocol was approved by the hospital’s ethical review board (Ethics Approval No. 2025-KL-343-02). All participants provided written informed consent prior to questionnaire completion and blood sample collection.

#### 2.1.2 Recruitment timeline and enrollment procedure

Enrollment was conducted continuously from May to June 2025 in Hangzhou (latitude ~30°N, late spring to early summer), with all venous blood sampling completed within this time window. This period was selected to minimize seasonal fluctuations in ambient ultraviolet radiation and serum 25(OH)D concentrations, as UV levels in Hangzhou remain relatively stable during late spring and early summer.

Participants were recruited via convenience sampling from Zhejiang Chinese Medical University. A self-designed online questionnaire was distributed via QR code on the SoJump survey platform^1^ to collect data on demographic characteristics, lifestyle habits, sun protection behaviors and skin color preferences. To ensure response authenticity, each IP address was limited to one submission, and all questions were required to be answered before final submission. Questionnaires completed in less than 3 min or with obvious regular response patterns were excluded from analysis. Pre-testing showed the formal questionnaire took approximately 10 min to complete. After questionnaire screening, all eligible participants attended the hospital’s health management center for fasting venous blood sampling.

#### 2.1.3 Sample size calculation and subgroup justification

Sample size estimation was conducted exclusively for the core cohort of regular university students, as all primary statistical inferences were restricted to this population. Calculations were based on the pre-experimental correlation coefficient between sun protection behavior scores and serum 25(OH)D concentrations (*r* = −0.223). With a two-tailed significance level of *α* = 0.05 and statistical power of 1−*β* = 0.80, a minimum of 156 valid participants was required to detect a statistically significant correlation. Accounting for a 10% anticipated rate of invalid questionnaires and participant attrition, we enrolled 174 regular university students, which fully met the statistical power requirements for all primary analyses.

Additionally, 17 students with regular outdoor physical training were recruited as an independent reference subgroup for descriptive comparison of vitamin D status distribution, to contextualize the impact of differing sunlight exposure levels among populations with comparable genetic backgrounds and skin phototypes. No formal inferential statistical tests were planned for this subgroup, so it was not subject to the same power calculation. Its relatively small size reflects the limited on-campus pool of eligible students with consistent long-term outdoor training regimens. This sample imbalance does not compromise core study conclusions, as all primary statistical inferences are derived solely from the adequately powered main cohort.

#### 2.1.4 Group setting and analytical strategy

Correlation tests and linear regression models included only data from the 174 regular university students, with no pooling of the two subgroups. Restricting core analyses to the regular student cohort minimizes confounding bias from population heterogeneity: this group has highly homogeneous baseline characteristics, including consistent daily academic schedules, campus residential environments, and general dietary patterns, which improves the internal validity of findings linking sun protection behaviors and skin tone aesthetic preferences to circulating 25(OH)D concentrations. The outdoor training subgroup serves exclusively as a descriptive reference to illustrate vitamin D level differences under disparate sunlight exposure patterns. The outdoor training subgroup serves exclusively as a descriptive reference only for simple between-group comparison of vitamin D status distribution and sun protection behavior prevalence. No skin tone preference, Tan Attractiveness Scale, bone turnover marker stratified analyses or cross-cultural aesthetic comparisons were performed on this subgroup; all descriptive and inferential statistical analyses in this manuscript were restricted solely to the 174 regular university students to maintain consistent sample eligibility. The outdoor-trained subgroup (*n* = 17) was excluded from all inferential statistical tests.

### 2.2 Measures

#### 2.2.1 Questionnaire development and validation

The self-designed questionnaire was developed and validated through a rigorous four-stage process. An initial pool of 35 items covering demographic characteristics, sun protection behaviors, skin tone preferences and lifestyle factors was first generated based on a systematic review of validated sun protection questionnaires and qualitative interviews with 10 university students to capture culturally specific sun protection practices in China. The initial draft was then reviewed by a panel of 5 experts (3 orthopedic surgeons specialized in bone metabolism, 1 public health epidemiologist, and 1 dermatologist), who rated each item for relevance, clarity and appropriateness on a 4-point scale (1 = irrelevant, 4 = highly relevant); item-level (I-CVI) and scale-level content validity index (S-CVI) were calculated thereafter, and items with I-CVI < 0.78 were revised or removed, yielding a final 28-item questionnaire with an overall S-CVI of 0.92, indicating excellent content validity. Finally, the revised questionnaire was pilot-tested in 30 university students not enrolled in the main study, after which minor revisions were made to improve item clarity and shorten the completion time to approximately 10 min, and the sun protection behavior subscale demonstrated good internal consistency with a Cronbach’s *α* coefficient of 0.79.

#### 2.2.2 Skin color preference assessment

**Figure 1:**
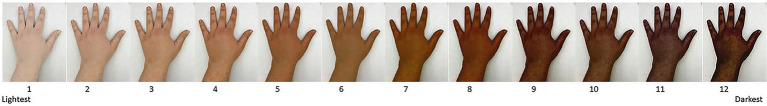
Skin tone spectrum scale for participant self-assessment of skin tone preferences. Reproduced from Sacksner et al. (2022), Archives of Dermatological Research, DOI: 10.1007/s00403-021-02320-0, with permission from Springer Nature (License No. 6311110412318). Copyright © 2022 Springer-Verlag GmbH Germany.

The validated skin color assessment scale originally created by Sackner et al. (12) was employed to evaluate individuals’ skin color preference (Figure 1). Participants selected their actual and ideal skin color options by referring to a set of hand skin tone images graded from level 1 (the lightest) to level 12 (the darkest). The skin tone difference value was computed using the formula of actual skin tone score minus ideal skin tone score, with a numerical range between −11 and 11. Specifically, a positive value represented the preference for fairer skin, whereas a negative value reflected the inclination toward darker skin tones.

#### 2.2.3 Tan Attractiveness Scale

The Tan Attractiveness Scale, a subdimension derived from the sun protection-related Final Appearance Motivation Attitudes scale established by Maddock et al. (13), was applied to evaluate participants’ perceptions of the attractiveness of tanned skin. This scale consists of 5 measurement items, with representative statements such as “Many people look much healthier with a tan” and “I look better with a tan.” All enrolled subjects indicated their level of concurrence with each item using a 5-point Likert-type response scale, with 1 corresponding to complete disagreement and 5 indicating complete agreement. Higher overall scores corresponded to a greater preference for tanned skin appearance. Reliability testing results revealed that the Cronbach’s *α* value of the Tan Attractiveness Scale reached 0.82 among the enrolled participants, suggesting satisfactory internal consistency and reliable psychometric properties for this scale.

#### 2.2.4 Sun protection behaviors assessment

Referring to existing relevant studies, participants were instructed to report the frequency of adopting three sun-protective behaviors when staying outdoors for over 1 h under strong sunlight: staying in shaded areas, applying sunscreen products, and wearing sun-protective clothing (14). Adoption frequencies for these three sun-protective practices were rated on a five-level scale: always, most of the time, sometimes, rarely, and never. These five response options were further grouped into three categories: regular (consistently and most occasions), moderate (at times), and rare (seldom and never). The composite sun protection behavior index was calculated by tallying the number of practices categorized as regular, with total scores ranging from 0 to 3. Subsequently, the number of sun protection approaches adopted by individuals was documented. Meanwhile, the overall score of the sun protection behavior scale was further computed. Each individual item was rated using a 5-point Likert scale where 1 indicated never and 5 indicated always, resulting in a cumulative scale score ranging between 3 and 15. We did not survey sunscreen SPF, application amount, exposed skin area, or environmental UV levels. Sun protection behavior patterns and daily sun exposure duration were used to evaluate participants’ overall sunlight exposure in statistical models.

#### 2.2.5 Covariates assessment

Referring to prior scholarly findings, this study incorporated confounding factors that have been proven to correlate with ultraviolet exposure habits and vitamin D insufficiency as covariates (15, 16). Participants were requested to fill out the basic information, including gender, age, nationality, residence type, education level, lifestyle (indoor or outdoor), monthly income, body mass index (BMI), and residential address. Participants were asked to report their daily durations of sun exposure, sedentary time, and sleep over the previous 30 days, stratified by workdays and non-workdays. Given the conventional weekly schedule consisting of five working days and two rest days, the average daily duration of each indicator was calculated following the formula below: Average daily minutes = (Workday minutes × 5 + Non-workday minutes × 2)/7. Data on consumption of foods naturally rich in vitamin D (including red meat, poultry eggs, fatty fish, dairy products), as well as similar items using the question: “How often do you eat?” The available response options were limited to two groups: ≥4 times and <4 times. Alcohol drinking status was defined as having alcoholic intake at least once per week within the past year. Smoking behavior was classified as a smoking history lasting 6 months or longer. The Short Form of the International Physical Activity Questionnaire (IPAQ-SF) was utilized to assess participants’ physical activity levels (17). Weekly metabolic equivalent of task (MET) minutes for walking, moderate-intensity exercise, and vigorous-intensity physical activity, along with overall weekly MET minutes, were computed in accordance with the standardized IPAQ scoring guidelines.

#### 2.2.6 Biochemical marker measurement

Upon completion of the questionnaires, all participants were instructed to visit the physical examination center of our hospital in a fasting state for venous blood sampling. Blood samples of 5 mL were drawn from all participants after at least 8 h of overnight fasting, with sampling conducted between 8:00 and 10:00 in the morning. All blood samples were centrifuged at 3000 rpm for 10 min at 4 °C within 30 min after collection to isolate serum. For samples not tested immediately, serum was aliquoted into sterile cryovials and stored at −80 °C until batch analysis. All samples underwent only one freeze–thaw cycle before biomarker detection to prevent analyte degradation. Serum indicators including circulating 25-hydroxyvitamin D [25(OH)D], type I procollagen N-terminal propeptide (PINP), β-cross-linked C-telopeptide of type I collagen (β-CTX), parathyroid hormone (PTH), osteocalcin (OC), and calcitonin (CT) were measured using electrochemiluminescence immunoassay (ECLIA). Detection was performed using a Cobas e 601 automatic analyzer (Roche Diagnostics, Mannheim, Germany) together with supporting commercial reagent kits. All laboratory technicians performing the biochemical assays were blinded to participants’ questionnaire responses, sun protection behavior classifications, and group assignments to minimize potential detection bias. All experimental operations were implemented strictly in line with the standard protocols provided by the manufacturer. The intra-assay coefficient of variation (CV) for all measured biomarkers ranged from 1.2–3.5%, while the between-assay CV ranged from 2.1 to 4.8%. These results verified that the adopted detection method possessed good reproducibility and stability.

### 2.3 Statistical analysis

This study adopted univariate linear regression to explore the correlations of skin color preference and sun protection habits with serum circulating 25(OH)D levels. All statistical analyses were performed using SPSS 26.0 statistical software (SPSS Inc., Chicago, IL, USA), and a two-sided *p*-value less than 0.05 was considered statistically significant. During data arrangement, any biochemical measurement results that fell below the assay’s detectable range were substituted with the minimum detectable concentration of the corresponding indicator. Continuous variables with normal distribution were expressed as mean ± standard deviation, while non-normally distributed data were presented as median and interquartile range. Categorical variables were described using frequencies and percentages. The Shapiro–Wilk test was employed to evaluate the normality of continuous data distributions. For within-cohort between-group comparisons of demographic characteristics among the 174 regular university students, the independent samples *t*-test, Mann–Whitney *U* test, and chi-square test were used as appropriate. No inferential tests were performed between regular students and outdoor-trained students. Linear regression models were constructed to examine the associations between sun protection behavior scores and skin tone scale scores with serum 25(OH)D concentrations. Multiple linear regression analysis was subsequently conducted after controlling for several potential confounding factors. In addition, the Bonferroni method was used to correct for type I error from multiple comparisons of bone turnover markers. For indicators with statistically significant overall intergroup differences, post-hoc pairwise comparisons with Bonferroni correction were further performed to identify the specific subgroup differences driving the significance.

## 3 Results

### 3.1 Baseline characteristics of the study population

Descriptive statistics for all study variables are presented in Table 1. This study recruited 174 young participants aged 20–29 years who were ordinary college students. Of these participants, 64 were male and 110 were female, representing 36.8 and 63.2% of the entire study population, respectively. The mean serum 25(OH)D level across all participants was 29.35 ± 11.82 nmol/L. In general, up to 93.1% (95%CI: 89.34, 96.87%) of individuals presented abnormal vitamin D insufficiency, defined as serum 25(OH)D concentrations <50 nmol/L. Specifically, 59.2% (95%CI: 51.89, 66.50%) suffered from vitamin D deficiency (<30 nmol/L) and 33.9% exhibited vitamin D insufficiency (30–49 nmol/L), while merely 6.9% (95%CI: 3.13, 10.66%) of participants achieved sufficient vitamin D levels (≥50 nmol/L). Additionally, nearly half of the respondents (53.5%) spent less than 30 min per day exposed to sunlight. Concerning sun-protective behaviors, 48.3% of participants engaged in 2–3 sun-protective practices, 84.5% frequently stayed in the shade, 24.7% frequently used sunscreen, and 41.4% frequently wore sun-protective clothing. In terms of skin tone preference, 64.4% desired lighter skin, while only 1.7% preferred darker skin. The mean score on the Tan Attractiveness Scale was 12.40 ± 3.30. More detailed demographic information of the enrolled participants is summarized in Table 1.

**Table 1:** Baseline characteristics of the study population (*n* = 174).

Category	Variables	*N* (%) or Mean ± SD
Demographic characteristics		
	Sex	
	Male	64(36.8)
	Female	110(63.2)
	Age (years)	24.48 ± 1.74
	Education level	
	Undergraduate degree	75 (43.1)
	Postgraduate degree and above	99 (56.9)
	Race	
	Han	167(96.0)
	Other	7(4.0)
	Residence	
	City	171 (98.3)
	Country	3 (1.7)
	Income level (RMB)	
	≤1999	121(69.5)
	2000~4,999	38 (22.0)
	5,000~9,999	13 (7.5)
	≥10,000	2 (1.0)
Lifestyle factors		
	Lifestyle	
	Indoors	163(93.7)
	Outdoors	11(6.3)
	Physical activity level	
	High level	15(8.6)
	Moderate level	92 (52.9)
	Low level	67 (38.5)
	Time spent outdoors (min/day)	28.27 ± 25.20
	Time spent outdoors (stratified)	
	<30	93 (53.5)
	30~59	72 (41.4)
	60~89	8 (4.5)
	90~119	1 (0.6)
	Sleeping time (h/day)	7.33 ± 0.69
	Sitting time (h/day)	7.80 ± 2.66
	Fish consumption	
	≥4 times	6 (3.5)
	<4 times	168 (96.5)
	Egg consumption	
	≥4 times	101 (58.0)
	<4 times	73 (42.0)
	Meat consumption	
	≥4 times	163 (93.7)
	<4 times	11 (6.3)
	Smoking behavior	
	Yes	3 (1.7)
	No	171 (98.3)
	Drinking behavior	
	Yes	6 (3.5)
	No	168 (96.5)
Exposure factors		
	Sun-protective behaviors level	
	0	15 (8.6)
	1	75 (43.1)
	2~3	84 (48.3)
	Staying in the shade	
	Rare	6 (3.4)
	Moderate	19 (10.9)
	Frequent	149 (85.6)
	Using sunscreen	
	Rare	78 (44.8)
	Moderate	53 (30.5)
	Frequent	43 (24.7)
	Wearing protective clothing	
	Rare	54 (31.0)
	Moderate	48 (27.6)
	Frequent	72 (41.4)
	Skin tone preference discrepancy (self-ideal)	
	Desired lighter skin (>0)	112 (64.4)
	Satisfied with skin (=0)	59 (33.9)
	Desired darker skin (<0)	3 (1.7)
	Ideal skin tone score	1.40 ± 0.66
	Score of Tan Attractiveness Scale	12.40 ± 3.30
Outcome indicators		
	Serum 25 (OH) D (nmol/L)	29.35 ± 11.82
	Vitamin D status	
	Deficiency (<30 nmol/L)	103 (59.2)
	Insufficiency (30 ~ 49 nmol/L)	59 (33.9)
	Sufficiency (≥50 nmol/L)	12 (6.9)

Values are expressed as mean + standard deviation (SD) or absolute (*n*) and relative (%) frequencies.

### 3.2 Distribution of vitamin D levels in the usual students and outdoor students

**Figure 2:**
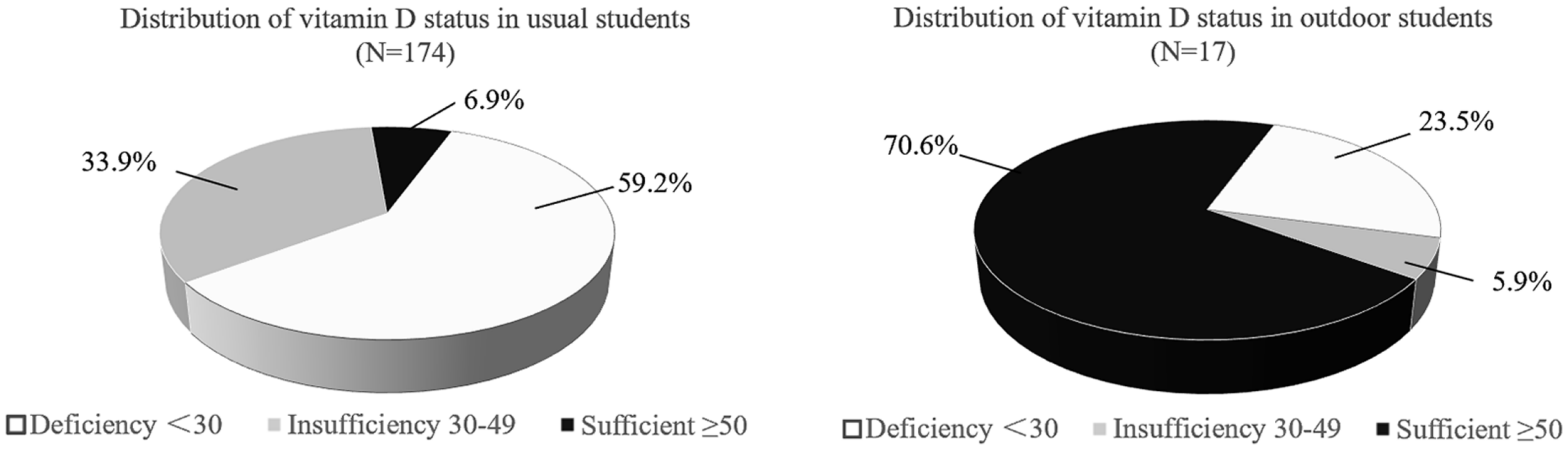
Distribution of vitamin D levels in the usual students and outdoor students.

The primary research subjects in the present study were general college students, with a total sample size of 174 individuals. The serum 25(OH)D distribution profile of this group is illustrated in Figure 2. Within this main cohort, as many as 93.1% (95%CI: 89.34, 96.87%) of subjects had 25(OH)D concentrations below 50 nmol/L. Further stratification indicated that 59.2% (95%CI: 51.89, 66.50%) were categorized as having vitamin D deficiency (<30 nmol/L), and another 33.9% (95%CI: 27.09, 41.27%) were classified as insufficient (30–49 nmol/L). A total of 17 outdoor sports students were enrolled as the control group for parallel comparison. In this control population, only 29.4% exhibited 25(OH)D concentrations below 50 nmol/L, consisting of 23.5% with deficiency and 5.9% with insufficiency. By contrast, 70.6% of outdoor participants maintained adequate vitamin D nutritional status (≥50 nmol/L). Visible disparities in vitamin D status stratification were observed between ordinary students and the control group. No formal statistical comparisons were performed for the outdoor subgroup due to its small sample size.

### 3.3 Characteristics of sun-protective behaviors in the usual students and outdoor students

**Figure 3:**
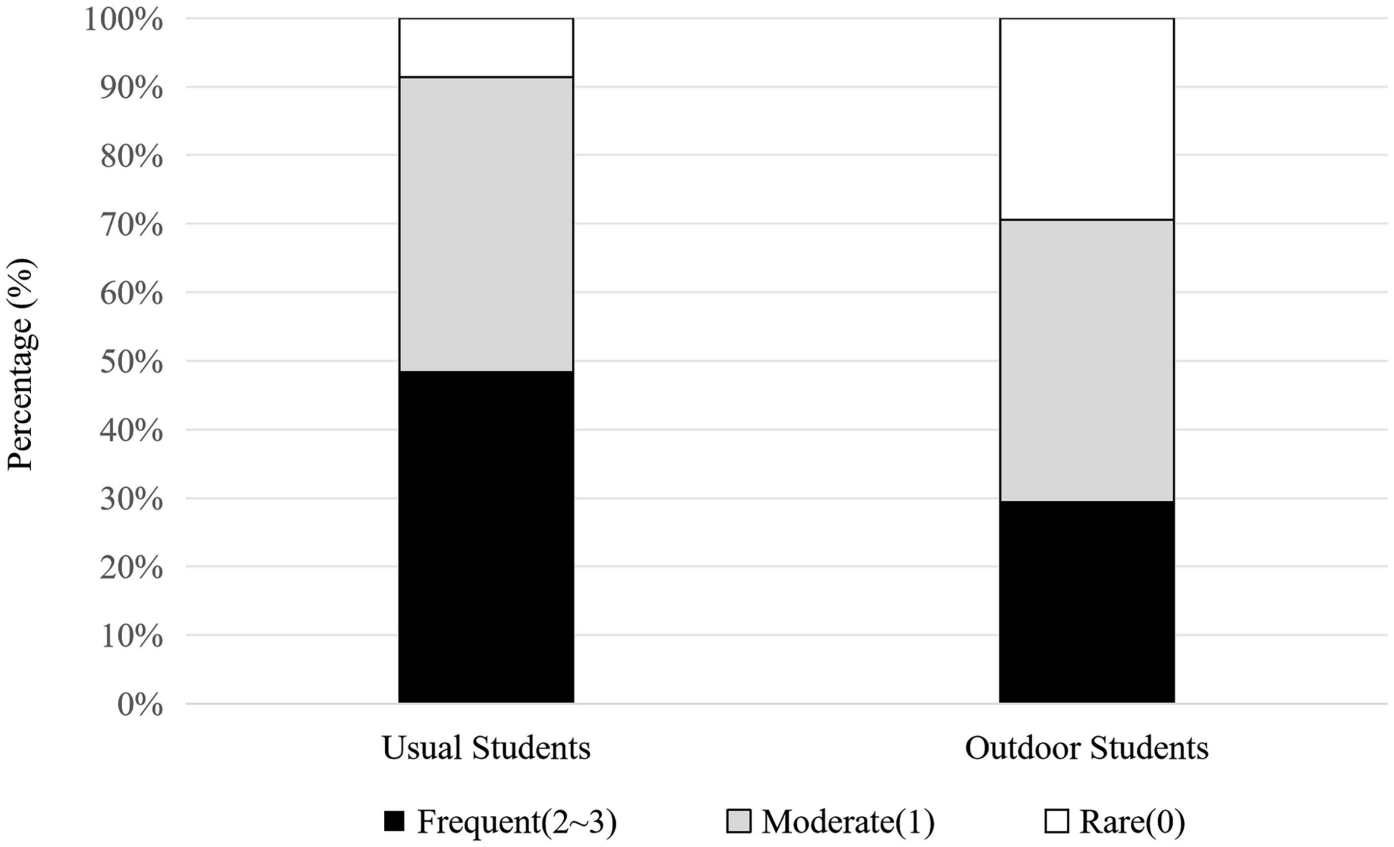
Distribution of sun protection levels in the usual students and outdoor students.

The core study population, usual students (*n* = 174), 91.4% of usual students used at least one sun-protective measure, with 43.1% adopting one single measure and 48.3% using 2–3 intensive concurrent modalities. Outdoor students (*n* = 17) served as a control group for comparative analysis (Figure 3). Usual students had a notably higher proportion of frequent intensive sun protection (48.3% vs. 29.4%) and a far lower proportion of rare/no sun protection (8.6% vs. 29.4%) than controls, while moderate sun protection rates were similar between groups.

### 3.4 Relationship between sun protection practices and circulating 25-hydroxyvitamin D concentrations

**Figure 4:**
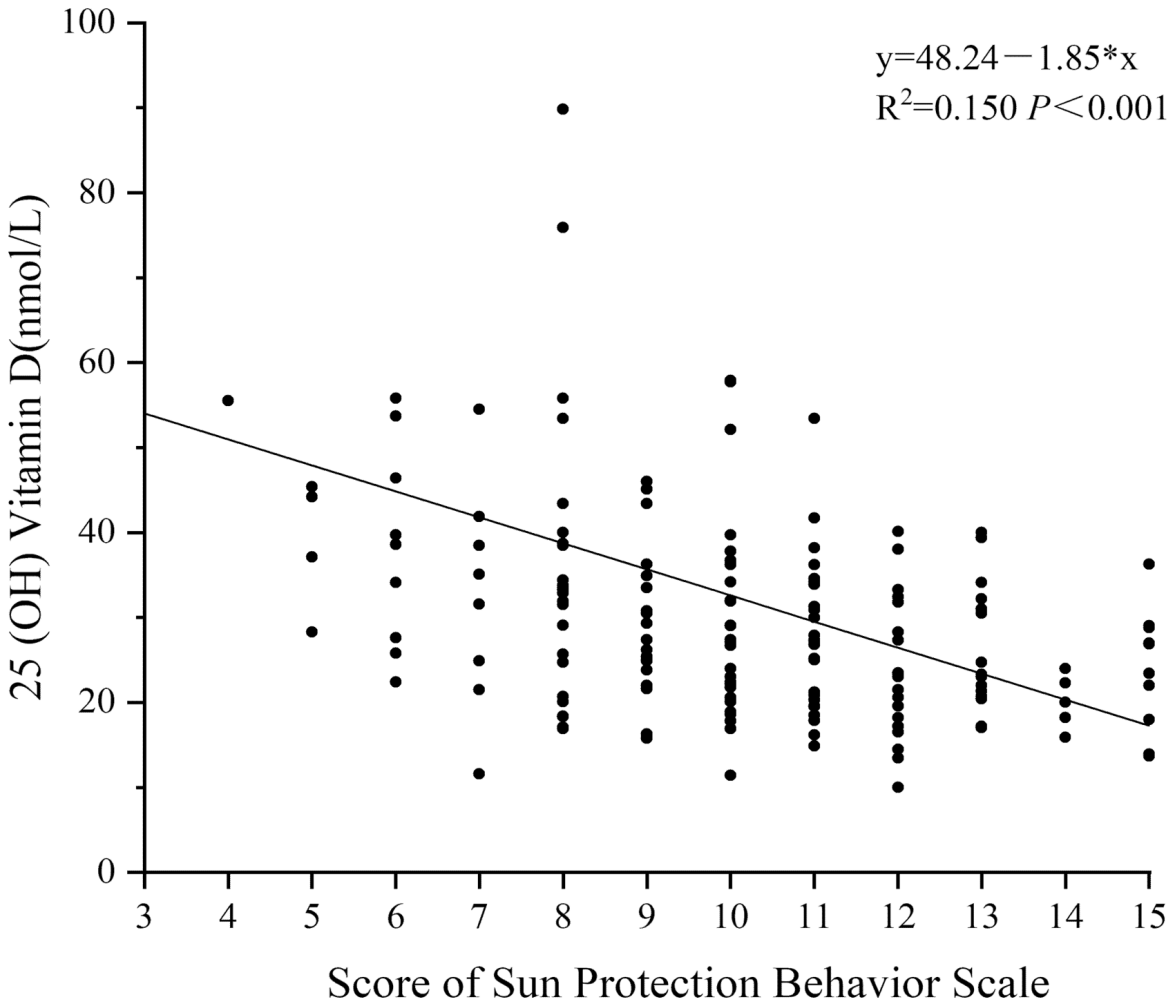
Linear regression analysis between sun protection behavior scale scores and 25(OH)D concentrations. Scatter plot showing the inverse linear correlation between sun protection behavior scale scores and serum 25(OH)D concentrations in 174 usual students. The 3–15 point scale quantifies the frequency of three core photoprotective practices, with higher scores indicating greater sun avoidance.

In the 174 regular university students, a significant inverse correlation was observed between sun protection behavior scores and circulating 25(OH)D levels (*r* = −0.387, *p* < 0.001), with the corresponding linear trend depicted in Figure 4. Table 2 summarizes the outcomes of three regression models exploring the link between sun protection behavior scores and serum 25-hydroxyvitamin D concentrations. The first model solely adjusted for age and gender; the second model additionally incorporated BMI and daily sunlight exposure duration. The third model further adjusted for physical activity intensity, as well as smoking and drinking habits. Across all three analytical frameworks, regression results consistently revealed a statistically significant inverse relationship between sun protection behavior scores and serum 25(OH)D. The standardized regression coefficients were −0.268 (*p* < 0.001) for Model 1, −0.241 (*p* = 0.003) for Model 2, and −0.217 (*p* = 0.007) for Model 3.

**Table 2:** Multiple linear regression analysis for vitamin D levels in relation to sun protection score among usual students after adjustment for potential confounders (*n* = 174).

Variable	Model 1				Model 2				Model 3			
	*B*	SE	*β*	*p*	*B*	SE	*β*	*p*	*B*	SE	*β*	*p*
Sun protection score	−1.285	0.379	−0.268	<0.001	−1.151	0.378	−0.241	0.003	−1.041	0.383	−0.217	0.007

*B*, unstandardized coefficients; SE, standard error; *β*, standardized coefficients. Model 1 was adjusted for age and gender; Model 2 was adjusted for age, gender, BMI, and daily sun exposure minutes; Model 3 was adjusted for age, gender, BMI, daily sun exposure minutes, physical activity energy level, and smoking/drinking status.

### 3.5 Associations of skin tone preference and tan attractiveness perception with sun-protective behaviors and vitamin D status

**Figure 5:**
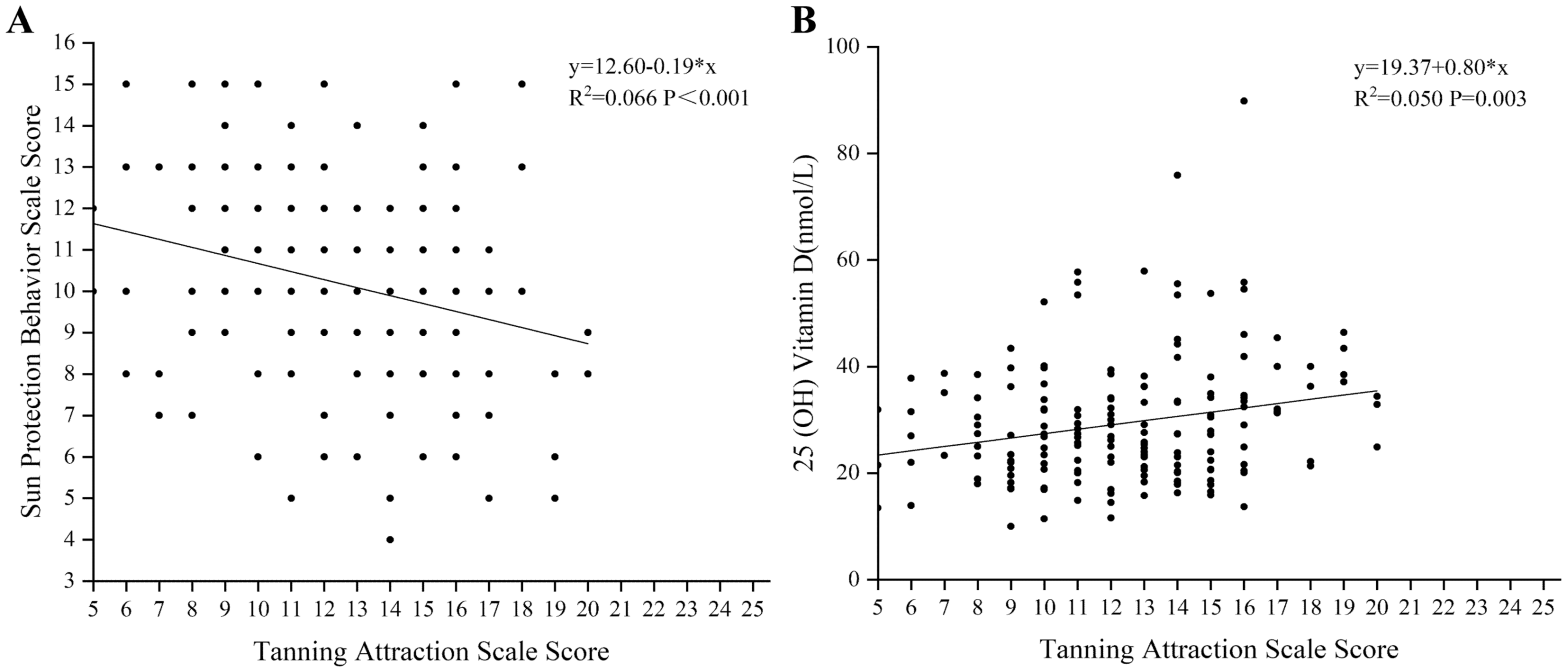
Linear regression of tanning attractiveness scale scores with sun protection behavior scores and serum 25-hydroxyvitamin D concentrations. Scatter plots showing linear associations between tanning attractiveness scale scores and sun protection behavior scores **(A)** Negative association: *y* = 12.60–0.19*x* (unstandardized *β* = −0.19, 95%CI: −0.30 to −0.08; *R*^2^ = 0.066, *p* < 0.001). **(B)** Positive association: *y* = 19.37 + 0.80*x* (unstandardized *β* = 0.80, 95%CI: 0.28 to 1.33; *R*^2^ = 0.050, *p* = 0.003). Higher scores indicate stronger preference for tanned skin; higher scores on the Sun Protection Behavior Scale indicate more frequent sun protection practices.

Scatter plot analyses revealed significant associations between tanning attraction scale scores and both sun protection behaviors and circulating 25-hydroxyvitamin D concentrations (Figure 5). Simple linear regression modeling demonstrated that tanning preference scores had a significant inverse association with sun protection behavior scores (unstandardized *β* coefficient = −0.19, 95% confidence interval: −0.30 to −0.08; *R*^2^ = 0.066, *p* < 0.001). The tanning attractiveness scale scores were positively associated with circulating 25(OH)D levels (*β* = 0.80, 95%CI: 0.28 to 1.33; *R*^2^ = 0.050, *p* = 0.003).

### 3.6 Cross-cultural descriptive comparison of skin tone aesthetic norms

A 12-shade skin tone spectrum (Figure 3, ranging from lightest shade 1 to darkest shade 12) was used to evaluate discrepancies between self-perceived and ideal skin tone. The mean skin tone discrepancy score was +0.8908 among regular university students (*n* = 174). Published data from 548 Western participants reported a mean skin tone discrepancy score of −0.0857 (12).

On the tanning attractiveness scale, the overall mean score for regular university students in our study (*n* = 174) was 12.40 ± 3.30, which was lower than the mean score of 15.80 ± 4.56 reported in a Western sample (*n* = 1,609) using the same scale (13). Stratified by gender, the mean scores were 12.64 ± 3.75 for Chinese males and 12.26 ± 3.01 for Chinese females, compared with 15.15 ± 4.92 for Western males and 16.18 ± 4.46 for Western females.

### 3.7 Relationships between sun protection practices, vitamin D nutritional status and bone turnover biomarkers

The associations between sun-protective practices with skin tone-related scale scores and bone turnover biomarkers among 174 regular university students are summarized in Table 3. Participants who adopted no sun protection measures had notably higher tanning attractiveness scores (14.53 ± 3.31) than those engaging in one protective behavior (12.41 ± 3.42) or two to three protective behaviors (12.00 ± 3.07), with statistically significant intergroup differences observed (*p* = 0.022). The preferred skin tone score was 1.67 ± 0.72 in the non-protection group, 1.56 ± 0.79 in the single protection group, and 1.20 ± 0.43 in the group with two to three protective behaviors (*p* < 0.001).

**Table 3:** Skin tone-related scores and bone turnover markers stratified by number of sun-protective behaviors among regular university students (*n* = 174).

Characteristics	All study participants (*n* = 174)	No. of sun-protective behaviors, *N* (%)			*p* value
		0 (*n* = 15)	1 (*n* = 75)	2–3 (*n* = 84)	
Tanning attraction scale score	12.40 ± 3.30	14.53 ± 3.31ᵃ	12.41 ± 3.42ᵇ	12.00 ± 3.07ᵇ	0.022
Number of favorite skin color	1.40 ± 0.66	1.67 ± 0.72ᵃ	1.56 ± 0.79ᵃᵇ	1.20 ± 0.43ᶜ	<0.001
25(OH)D (nmol/L)	27.30 (20.97, 34.35)	31.50 (25.35, 44.80)ᵃ	30.50 (22.30, 38.50)ᵇ	23.45 (19.60, 31.07)ᶜ	<0.001
PTH (pmol/L)	3.79 (3.10, 4.71)	3.46 (3.08, 4.08)ᵃ	3.50 (2.88, 4.64)ᵃᵇ	4.70 (3.47, 4.95)ᵇ	0.043
β-CTX (ng/L)	525.0 (406.2, 622.8)	525.0 (457.5, 768.5)ᵃ	572.0 (423.0, 762.5)ᵇ	483.5 (383.8, 593.8)ᶜ	0.004
PINP (μg/L)	51.95 (44.35, 65.05)	64.90 (48.70, 86.20)ᵃ	56.20 (47.00,69.35)ᵇ	48.90 (41.30, 59.32)ᶜ	<0.001
OC (μg/L)	19.60 (16.60, 23.35)	22.60 (17.30, 27.85)ᵃ	20.40 (16.80, 26.10)ᵇ	18.80 (15.78, 22.55)ᵇ	0.023
CT (pmol/L)	0.40 (0.15, 0.87)	0.20 (0.07, 0.54)ᵃ	0.43 (0.21, 0.96)ᵇ	0.37 (0.07, 0.90)ᵃᵇ	0.075

Values are presented as mean ± standard deviation or median (interquartile range) as appropriate. All analyses in this table are limited to the 174 regular university students; outdoor-trained students (*n* = 17) were excluded. Within each row, values with different superscript letters (a, b, c) are significantly different at *p* < 0.05 after Bonferroni correction. *p* values in the last column are derived from one-way ANOVA (for normally distributed variables) or Kruskal–Wallis test (for non-normally distributed variables) for overall comparisons across the three groups. Post-hoc pairwise comparisons were performed using Bonferroni test (for ANOVA) or Dunn’s test with Bonferroni correction (for Kruskal–Wallis test). 25(OH)D, 25-hydroxyvitamin D; PTH, parathyroid hormone; B-CTX, C-terminal telopeptide of type I collagen; PINP, procollagen type I N-terminal propeptide; OC, osteocalcin; CT, calcitonin.

Median 25(OH)D concentrations were 31.50 (25.35, 44.80) nmol/L in participants with no sun protection measures, 30.50 (22.30, 38.50) nmol/L in those with one protective behavior, and 23.45 (19.60, 31.07) nmol/L in those with two to three protective behaviors (*p* < 0.001). PTH levels were 3.46 (3.08, 4.08) pmol/L in the no-sun-protection group, 3.50 (2.88, 4.64) pmol/L in the single protection group, and 4.70 (3.47, 4.95) pmol/L in the group with two to three protective behaviors (*p* = 0.043). The bone resorption marker β-CTX was 525.0 (457.5, 768.5) ng/L in the no-protection group, 572.0 (423.0, 762.5) ng/L in the single protection group, and 483.5 (383.8, 593.8) ng/L in the two to three protection group (*p* = 0.004). For bone formation markers, PINP concentrations were 64.90 (48.70, 86.20) μg/L, 56.20 (47.00, 69.35) μg/L and 48.90 (41.30, 59.32) μg/L across the three groups, respectively, (*p* < 0.001). OC levels were 22.60 (17.30, 27.85) μg/L, 20.40 (16.80, 26.10) μg/L and 18.80 (15.78, 22.55) μg/L (*p* = 0.023). Calcitonin (CT) concentrations were 0.20 (0.07, 0.54) pmol/L in the no-sun-protection group, 0.43 (0.21, 0.96) pmol/L in the single protection group and 0.37 (0.07, 0.90) pmol/L in the group with two to three protective behaviors (*p* = 0.075).

## 4 Discussion

The present observational cross-sectional investigation enrolled 174 young Chinese university students aged 20–29 years in Hangzhou, aiming to explore their circulating 25-hydroxyvitamin D concentrations and their associations with sun protection behaviors shaped by cultural perceptions. With regard to the empirical findings of our study, the findings demonstrated an extremely alarmingly high prevalence of suboptimal vitamin D status (93.1%) and frank vitamin D deficiency (59.2%) among this young student population. In addition, the study participants were well-educated urban young adults with unimpeded access to nutrient-rich foods, yet their vitamin D status was still severely suboptimal. A descriptive disparity in vitamin D status between usual college students (6.9% sufficient) and outdoor students (70.6% sufficient) among the same ethnic group was also observed.

In line with previous epidemiological evidence, similar findings have been reported in previous studies (18, 19). These figures are not only far higher than the prevalence reported in Western adult populations, but also highlight a severe vitamin D nutritional gap among young, highly educated urban Chinese individuals (20). For young Chinese adults, sunlight exposure is still the primary pathway for endogenous vitamin D production, whereas regular dietary intake and unspecific nutritional supplementation exert only a limited influence on circulating 25(OH)D concentrations (21). A descriptive disparity in vitamin D status between usual college students (6.9% sufficient) and outdoor students (70.6% sufficient) among the same ethnic group tentatively suggests the irreplaceable role of adequate sun exposure in maintaining vitamin D homeostasis. Notably, this intergroup comparison is only a descriptive observation based on merely 17 outdoor students; the small sample lacks sufficient statistical power, and the observed difference cannot be generalized or considered robust comparative evidence. These descriptive observations tentatively indicate that sufficient sunlight exposure serves as the primary and most effective means to sustain favorable vitamin D status among individuals with accessible outdoor activities, particularly young adults (22), noting that uncollected vitamin D supplement data may bias this tentative association. Taken together, these observations offer a tentative interpretation that all participants shared similar genetic backgrounds and skin pigmentation, yet the lack of vitamin D supplement records prevents definitive causal inference that the high prevalence of vitamin D insufficiency among general university students is closely related to insufficient daily sunlight exposure.

Endogenous vitamin D synthesis relies on cutaneous UVB exposure, which is effectively inhibited by sunscreen use, protective clothing, and shade-seeking (23). In our cohort, this study found that 91.4% of usual students adopted at least one sun-protective measure, and 48.3% used two or three concurrently. The between-group differences in sun protection behaviors were consistent with the distribution of vitamin D levels. Frequent and multiple sun protection practices were closely correlated with poor vitamin D status in university students. Our analysis identified a strong negative correlation between sun protection behavior scores and circulating 25-hydroxyvitamin D concentrations (Pearson correlation coefficient *r* = −0.387, *p* < 0.001). Linear regression further validated a distinct dose–response pattern: incremental rises in sun protection scores were accompanied by significant reductions in circulating 25(OH)D concentrations, and this independent relationship remained robust even after controlling for age, sex, body mass index, daily sun exposure time, and lifestyle-related confounding factors. The multiple linear regression models consistently confirmed this inverse association after adjusting for multiple confounders, revealing that increased sun protection was independently associated with lower serum 25(OH)D levels. We further identified a dose–response relationship between sun-protective behavior frequency and BTMs. PTH levels increased progressively with more frequent sun protection, consistent with compensatory secondary hyperparathyroidism in the setting of lower vitamin D status; meanwhile, bone turnover markers (β-CTX, PINP, OC) were significantly higher in participants with fewer protective behaviors. This observation may initially appear paradoxical relative to the classic model of vitamin D deficiency–driven secondary hyperparathyroidism with high bone turnover, but it aligns with the multifactorial regulation of bone remodeling in this young population. Bone turnover is co-modulated by mechanical loading and endocrine signals (24). Participants with minimal sun protection had greater outdoor weight-bearing activity; the pro-remodeling effect of mechanical stimulation outweighed the mild compensatory PTH elevation associated with lower vitamin D status. Notably, PTH levels across all groups remained within the physiological range, reflecting only mild compensation rather than pathological hyperparathyroidism sufficient to drive elevated bone turnover (25, 26). In the high sun protection group, reduced mechanical loading suppressed baseline bone turnover to a degree that exceeded the mild stimulatory effect of compensatory PTH elevation, explaining the observed pattern.

When compared with existing literature and established biological mechanisms, this protective pattern is far more intensive than that reported in U.S. adults, where only a minority adopt multiple sun protection strategies (14). This quantitative dose–response relationship indicates a strong negative association between intensive sun protection and cutaneous vitamin D synthesis, in agreement with previous epidemiological findings (27). These results are in accordance with the well-established biological function of vitamin D in regulating calcium balance and bone metabolism (28). Collectively, these data are consistent with the hypothesis that the observed gradient in PTH levels aligns with compensatory secondary hyperparathyroidism in the setting of lower vitamin D status, and the differences in bone turnover markers align with greater bone remodeling activity in the context of higher vitamin D availability. This trend is consistent with the proposed mechanism that sun protection may influence cutaneous vitamin D synthesis, which may in turn be associated with altered skeletal metabolic profiles, though a causal direction cannot be established in the present cross-sectional design.

Turning to skin tone aesthetic preferences in our study population, our data showed that 64.4% of participants preferred lighter skin, while only 1.7% favored a darker complexion (Table 1), with a mean Tan Attractiveness Scale score of 12.40 ± 3.30. Participants with a stronger preference for tanned skin tended to adopt fewer sun protection measures. Scatter plot combined with regression analyses further verified that higher tanning attractiveness perceptions were linked to lower engagement in sun-protective practices and elevated serum 25(OH)D levels (Figure 5). We further conducted a descriptive cross-cultural comparison of skin tone aesthetic norms. Using a 12-grade skin tone spectrum, the mean skin tone discrepancy score was positive among Chinese participants. In comparison with published data from Western populations, the mean tan attractiveness score was significantly lower than published values in Western populations, reflecting a weak preference for tanned skin among young Chinese college students (13). In contrast, Western participants presented a slightly negative discrepancy score, indicating a general preference for tanned skin (12). This finding is consistent with prior evidence that sociocultural values and ideal skin tone shape sun-related behaviors in Asian populations (29). In East Asian societies, fair skin has traditionally symbolized health, beauty, and social status, driving persistent sun avoidance starting in adolescence (30, 31). Taken together, these findings suggest that cultural aesthetic preferences are associated with sun avoidance behaviors, consistent with the proposed sociocultural pathway linking skin tone ideals to photoprotection practices. Accordingly, individuals with more positive attitudes toward tanning had higher circulating 25(OH)D concentrations, which may be partially explained by greater sunlight exposure and less frequent photoprotection in this group. This creates a notable tension between cultural aesthetic norms and skeletal health: intensive photoprotection driven by fair-skin ideals is associated with lower circulating 25(OH)D concentrations, which may have implications for bone health during the critical stage of peak bone mass accumulation (32). Although the tanning attractiveness score only explained 5–6% of the variance in sun protection behaviors and vitamin D levels, this result is still of statistical and public health significance, as cultural aesthetic orientation has been proposed as an upstream sociocultural correlate of sun protection behaviors.

The low variance explanation rate may be due to the fact that sun protection behaviors are also influenced by other factors, such as popular science of sun protection, social media appearance propaganda, and personal skin sensitivity (29). Such opposite tendencies in skin tone preference between the two populations further explain the differences in sun-related behaviors across cultures. In addition, we did not perform direct statistical comparisons between our data and previous Western studies due to differences in study design, recruitment strategies and population characteristics. Thus, the results of this cross-cultural descriptive comparison should be interpreted with caution.

The present findings hold valuable implications for clinical practice and public health intervention in China. The cultural pursuit of fair skin in this societal context coincides with an alarmingly high rate of vitamin D insufficiency in young adults, lending important practical and population-level reference significance (33, 34). Existing sun safety promotion initiatives in China primarily center on skin cancer prevention, while largely neglecting the associated risk of vitamin D insufficiency. This represents a notable policy gap, particularly considering China’s relatively low skin cancer incidence alongside the highly prevalent suboptimal vitamin D status among the general population (35). Our findings highlight an important public health gap: current domestic sun protection promotion mainly focuses on skin damage prevention while ignoring the risk of vitamin D insufficiency among young Chinese students. Consistent with our observation that culture-driven heavy sun avoidance strongly correlates with poor vitamin D status, targeted health education is urgently needed for young adults to raise awareness of the skeletal risks brought by excessive sun blocking. Future public health campaigns should deliver balanced messages that reconcile the cultural preference for fair skin with bone health maintenance, reminding the public that extreme long-term sun avoidance may impair vitamin D synthesis and peak bone mass accumulation.

This study is subject to certain inherent limitations. First, single-center convenience sampling in Hangzhou limits the generalizability of our results to rural populations and high-latitude areas with low UV exposure. Specifically, our findings are derived from university students in a single subtropical city, and thus cannot be directly generalized to young adults in other geographic regions, educational settings or population groups. Randomized multicenter research is necessary to replicate these findings. Second, sun-protective behaviors and skin tone preference were evaluated using self-reported questionnaires, which are prone to recall inaccuracies and social desirability bias. Third, the small sample of 17 outdoor students may compromise the statistical power of group comparisons. Owing to the limited sample size and insufficient statistical power for formal inferential testing, no comparative statistical analyses were performed for this subgroup, and its data were used solely for descriptive reference. Accordingly, the between-group differences observed should be interpreted with caution. Fourth, vitamin D supplement intake data were not captured, representing a major unmeasured confounder for 25(OH)D. Supplement use among urban university students may alter vitamin levels independently of sun exposure, introducing unquantifiable residual confounding. Imbalanced supplement use across sun-protection groups may distort the observed correlation, limiting the robustness of our regression findings. Finally, detailed indicators including sunscreen parameters, exposed skin area and environmental UV levels were not collected. We adopted sun protection behaviors and daily sun exposure duration as alternative indicators to assess sunlight exposure.

Notwithstanding the above limitations, this study yields novel and robust empirical evidence demonstrating a relationship between culturally driven sun protection practices and vitamin D status as well as bone metabolic profiles in young Chinese college students. The quantitative dose-effect estimate of the sun protection-vitamin D relationship adds to the evidence base for East Asian populations (36). Healthcare providers and public health programs should raise awareness of the potential risks of excessive sun avoidance, particularly within the prevailing sun-protective culture in China. Targeted public education regarding vitamin D nutritional status and appropriate sun exposure is critically required for young adults during the crucial phase of peak bone mass acquisition (37).

## 5 Conclusion

Our current study found that young urban Chinese undergraduates exhibit poor serum 25(OH)D profiles, alongside a concerningly large proportion experiencing insufficient or deficient vitamin D levels. Due to missing data on vitamin D supplementation, causal links cannot be firmly established; this descriptive phenomenon appears largely linked to widespread sun avoidance habits shaped by societal ideals favoring fair complexion, which in turn limits regular outdoor light exposure in this student group. It is therefore essential to formulate culturally compatible intervention measures to help enhance vitamin D nutritional levels among Chinese college students. Health initiatives ought to advocate appropriate, non-burning outdoor sunlight exposure and increased consumption of vitamin-rich foods, while discouraging excessive sun-protective behaviors to lower the likelihood of vitamin D depletion. Public health authorities are advised to develop rational UV protection guidelines that properly balance mainstream aesthetic values and skeletal health demands. Additionally, additional studies are required to examine the practicality of implementing individualized vitamin D supplementation regimens and routine biochemical monitoring for those young individuals who persistently avoid sunlight purely for cosmetic purposes, so as to sustain their vitamin D sufficiency over time.

## Data Availability

The raw data supporting the conclusions of this article will be made available by the authors, without undue reservation.

## Footnotes

1
https://www.wjx.cn
